# Diversity, Abundance and Community Structure of Benthic Macro- and Megafauna on the Beaufort Shelf and Slope

**DOI:** 10.1371/journal.pone.0101556

**Published:** 2014-07-09

**Authors:** Jessica Nephin, S. Kim Juniper, Philippe Archambault

**Affiliations:** 1 School of Earth and Ocean Sciences, University of Victoria, Victoria, British Columbia, Canada; 2 Institut des Sciences de la Mer de Rimouski, Université du Québec à Rimouski, Rimouski, Québec, Canada; University of Tasmania, Australia

## Abstract

Diversity and community patterns of macro- and megafauna were compared on the Canadian Beaufort shelf and slope. Faunal sampling collected 247 taxa from 48 stations with box core and trawl gear over the summers of 2009–2011 between 50 and 1,000 m in depth. Of the 80 macrofaunal and 167 megafaunal taxa, 23% were uniques, present at only one station. Rare taxa were found to increase proportional to total taxa richness and differ between the shelf (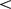 100 m) where they tended to be sparse and the slope where they were relatively abundant. The macrofauna principally comprised polychaetes with nephtyid polychaetes dominant on the shelf and maldanid polychaetes (up to 92% in relative abundance/station) dominant on the slope. The megafauna principally comprised echinoderms with *Ophiocten* sp. (up to 90% in relative abundance/station) dominant on the shelf and *Ophiopleura* sp. dominant on the slope. Macro- and megafauna had divergent patterns of abundance, taxa richness (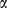 diversity) and 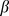 diversity. A greater degree of macrofaunal than megafaunal variation in abundance, richness and 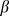 diversity was explained by confounding factors: location (east-west), sampling year and the timing of sampling with respect to sea-ice conditions. Change in megafaunal abundance, richness and 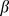 diversity was greatest across the depth gradient, with total abundance and richness elevated on the shelf compared to the slope. We conclude that megafaunal slope taxa were differentiated from shelf taxa, as faunal replacement not nestedness appears to be the main driver of megafaunal 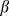 diversity across the depth gradient.

## Introduction

In the Arctic, the pace of climate warming is accelerated, compared to other regions [1], exposing areas like the Canadian Beaufort Shelf to new pressures such as shipping traffic, exotic species, oil and gas extraction and possibly commercial fishing. Arctic marine benthos, which provide key ecosystem functions such as nutrient cycling, organic matter transport, sediment mixing and metabolization of pollutants [2] will likely be influenced by many of the direct and indirect effects of climatic driven changes [3].

The effect of a longer ice-free season on the benthos is currently under debate [4], [5]. Thinning and reduced ice conditions accompanied by upwelling favourable winds [6] may increase primary productivity and the benthic standing stock [7]–[9]. Alternatively, the loss of sinking ice-algae and a shift toward open-water primary productivity may lead to a zooplankton-dominated ecosystem and a decrease of food supply for the benthos [10], [11]. In addition, warming Arctic seas may facilitate changes in benthic community structure through the introduction of lower latitude taxa [11]–[13]. Hence, the fate of Arctic shelf benthos and the tightly coupled pelagic environment [14] in a continuing climate warming scenario remains unclear. Recently, a renewed interest in industrial exploration of the Canadian Beaufort Sea has prompted a resurgence of benthic surveys providing a baseline for which to monitor future change. Understanding regional spatial patterns and drivers of benthic abundance and diversity is needed to effectively monitor potential human induced shifts [3].

On continental margins benthic patterns principally vary across the depth gradient [15]. There is wide acceptance that continental shelf benthos decrease in abundance with increasing depth [16] as a result of decreases in the flux of particulate organic carbon on which they rely [17], [18]. Patterns of benthic taxa richness across depth gradients are less consistent [19], [20], although theory predicts a unimodal distribution with peak diversity occurring at mid-slope where shallow and deep-sea species ranges overlap [15], [21]. In the Arctic, macro- and megafaunal abundance and taxa richness are observed to decrease monotonically with depth from mid-shelf to slope [14], [22], [23] as does the flux of particulate organic matter [24]. However, few marine studies have examined the contribution of rare species to local species richness [25], [26] and how the distribution of rare species may vary with depth. Factors that affect the distribution of rare species may be important for monitoring and conservation, as rare species are theorized to buffer against alterations in ecosystem function under environmental change, even those functionally similar to dominants [26]–[28].

Benthic community composition also varies across the depth gradient. Previous work in the Canadian Beaufort has shown macrofauna composition to be similar at corresponding depths along the shelf [23]. This observation is consistent with the expectation of faunal replacement (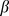 diversity [29]) across the bathymetric gradient, largely in response to decreased food availability [17]. However, Arctic benthos have been predicted to have larger depth ranges and thus display a slower rate of faunal replacement across depth gradients [17]. On the pan-Arctic scale, there is evidence of large overlap between shelf and slope taxa, suggesting that many taxa may be eurybathic [22], [30] and that the slope benthos is simply a nested sub-assemblage of shelf benthos rather than being a community that replaces the shelf fauna as depth increases or food supply diminishes. The distinction between spatial replacement and nested structure may be important to understanding how present day food availability is determining faunal distribution patterns and the response of benthos to predicted changes in future food availability. Furthermore, several studies have demonstrated that 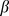 diversity (faunal replacement) can vary between faunal groups [31]–[34], likely due to differences in metabolism, trophic structure, mobility and dispersal [15]. The degree to which the rate of faunal replacement differs between Arctic macro- and megafauna has yet to be quantified.

To inform future monitoring programs on the Canadian Beaufort Shelf we compared macro- and megafaunal patterns of rarity, abundance and community composition. Specifically our objectives were to determine: 1) what factors co-vary with the distribution of rare taxa, and the similarity of macro- and megafaunal patterns of 2) abundance and taxa richness (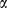 diversity) and 3) 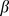 diversity.

## Methods

### Study Area

**Figure 1:**
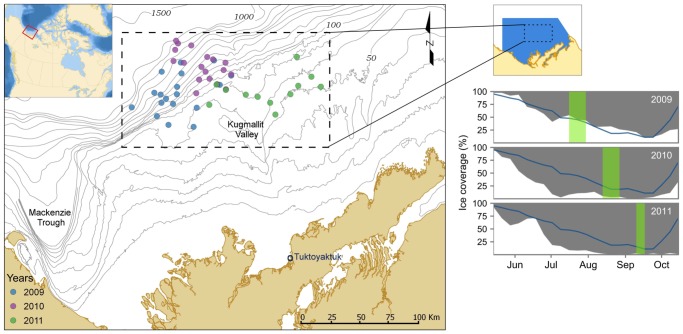
Sampling stations and ice coverage on the Beaufort shelf and slope from 2009 to 2011. One box core and trawl sample were collected from each station (left panel). Sample sizes were n = 18 in 2009, n = 18 in 2010 and n = 12 in 2011. Black dotted line outlines the spatial extent of sampling used to calculate the average slope. Ice coverage (white area, right panel) for 2009, 2010 and 2011 benthic sampling periods. Blue coverage area outlines the area over which ice coverage was calculated. Blue lines in plots represent historic ice coverage (median from 1981 to 2010). Green bars indicate when benthic sampling occurred. Ice coverage data courtesy of Canadian Ice Service, Environment Canada.

The Canadian Beaufort Shelf is a long and narrow (450 km by 130 km) Arctic shelf covering approximately 64,000 km^2^ (Figure 1). Both the shelf and adjoining slope study areas are bounded by the Mackenzie Trough to the west, the Mackenzie Delta to the south and the Amundsen Gulf to the east. The slope begins between the 80 m and 200 m isobaths [35], [36]. Within the spatial extent of this study area (shown by the dotted black line in Figure 1) the shelf break was located at 100 meters in depth. The shelf break was defined as the depth at which the rate of change of the average slope, modelled by the logistic function: 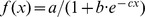 where 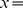 depth, was the greatest. The continental slope gradually drops in depth at an angle of 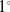 to 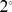 between the shelf break and 1000 m. Within the study area, the depth range of the shelf is 50 m (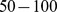 m) and depth range of the slope is 900 m (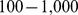 m).

The dominant bottom current moves water of both Pacific (surface) and Atlantic (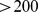 m) [9], [37] origin eastward along the shelf and slope [35], [36]. Upwelling can occur all along the shelf break bringing nutrient rich water onto the shelf [38]. Several features enhance upwelling of deep water on the Beaufort Shelf: the wide and deep Mackenzie Trough [39], the narrow and stable Kugmallit Valley [38], [40] and the near shore steep slope east of Cape Bathurst [41], [42].

### Sampling

Benthic sampling was undertaken through a partnership between ArcticNet (www.arcticnet.ulaval.ca), British Petroleum, Imperial Oil and the Canadian Healthy Oceans Network [43] to gather baseline benthic data in the oil and gas exploration lease areas of the Beaufort shelf and slope. Samples were collected within a 12,000 square kilometre spatial extent, northeast of the Mackenzie Trough (Figure 1). Sampling occurred during three summer expeditions on the CCGS Amundsen during the 2009, 2010 and 2011 ArcticNet field programs. Data from 48 sampling stations within Imperial Oil's and British Petroleum's exploration license areas (Ajurak, Pokak, EL451 and EL453) were utilized in this study.

At each sampling station, macrofauna were sampled using a 0.25 m^2^ USNEL box corer and megafauna were sampled with an Agassiz trawl (1.5 m in width, 0.7 m in height). On average the 48 paired box core and trawl samples were separated by 770 m (range 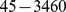 m) in horizontal distance and 7 m (range 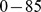 m) in depth. Sediment from half of the surface area of the box corer was utilized down to a maximum depth of 15 cm. The surface area sampled was 0.125 m^2^ and the average volume sampled was 1200 cm^3^. Macrofauna were collected on a 0.5 mm mesh sieve and fixed in 4% buffered formalin for later identification. Towing speed for trawls ranged from 1.5 to 2 knots and bottom time from 3 to 5 minutes, with the exception of the 2009 trawls where bottom time was 10 minutes. The trawl mesh was 5 mm and samples were sieved with a 2 mm mesh after collection, with the exception of 2009 sampling where a 0.5 mm sieve was used. Faunal densities were standardized to the average trawl area: 450 m^2^ (trawl net width 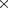 ship speed 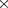 bottom time). Megafauna which could not be confidently identified onboard were preserved in 4% buffered formalin or frozen at 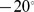 Celsius. Megafauna identified onboard were discarded or used for other analyses.

Benthic sampling was not consistent between sampling years; samples were distributed asymmetrically between shelf and slope and with each subsequent year were taken later in the summer season and farther to the east (Figure 1). In addition, sea ice conditions in the Beaufort varied considerably during these years. The sea-ice breakup on the Beaufort shelf was earlier in the year and reached a lower minimum ice coverage in 2010 and 2011 (Figure 1).

### Data preparation and quality control

All benthic samples were collected, processed and identified to the lowest taxonomic level possible using the same protocol across all sampling years. The metadata can be accessed through the Polar Data Catalogue (www.polardata.ca) and datasets will be publicly accessible through Dryad (datadryad.org). The resulting faunal datasets needed some modifications prior to use in this study, primarily to ensure the consistent use of taxonomic names––in order to prevent the inflation of taxa richness. Both box core and trawl datasets were validated through the removal of synonyms and unaccepted names using the WoRMS (www.marinespecies.org) Taxon Match tool.

Only 46% of box core and 60% of trawl faunal specimens were identified to the species level. The majority of these higher-order identifications were the result of broken or damaged specimens and the lack of taxonomic focus or expertise within certain phyla such as Sipuncula and Nemertea. Excluding all higher-order taxa to standardize the data to the species level would remove too large a portion of the total records. Alternatively, specimens consistently identified to higher-orders (e.g. Nemertea) remained in the database while specimens identified to several taxonomic ranks (e.g. Ophiuridae (Family), Ophiurinae (Subfamily), *Ophiocten* (Genus), *sericeum* (species)) were grouped to the family level. Records were removed from the database only if specimens that were identified to several taxonomic ranks were ranked higher than the family level (e.g. Ophiurida (Order)). This system was employed to balance the retention of detail and the loss of records from the dataset. The resulting datasets included 73% of box core records and 92% of trawl records. Grouping organisms identified to several taxonomic ranks acts as a quality control mechanism by minimizing any potential interannual variability in taxonomic identifications. Previous studies have validated a higher-taxa approach to data quality control by demonstrating that grouping taxa into higher taxonomic classes has little effect on the detection of diversity patterns [30], [44], [45].

The box core and trawl tended to selectively sample macrofauna and megafauna, respectively. Seventy-nine taxa (32%) were sampled by both gear types. However, the shared taxa were not sampled in a quantitatively comparable way by the two gear types. The trawl, because of its limited penetration of the sediment and larger mesh size, would tend to undersample the macrofauna. On the other hand, the box corer would tend to inaccurately sample the more widely spaced megafaunal organisms, because of its relatively small surface area. Two distinct quantitative datasets were created by removing macrofauna from the trawl samples and megafauna from the box corer samples. Taxa were identified as macrofauna or megafauna based on the frequency at which they were sampled by each gear type, assuming that megafauna were collected more frequently and effectively by the trawl than by the box corer and vice versa. The removal of shared taxa resulted in a 40% reduction in box corer taxa and a 30% reduction in trawl taxa. In addition, meiofauna and colonial fauna were removed from the datasets; meiofauna are not consistently sampled with larger mesh sieves and colonial fauna are not suitable for individual count data.

### Analyses

All statistical analyses were completed in the R environment for statistical computing (www.r-project.org) with aid from community ecology and graphics packages: vegan, cluster, rich and ggplot. Maps and spatial analyses were completed using QGIS software (http://qgis.osgeo.org).

Total abundance (number of individuals) was calculated based on the standardized average sample (0.125 m^2^ for macrofauna and 450 m^2^ for megafauna). Spearman's rank correlation (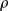) was used to quantify the strength of abundance and occupancy trends. Occupancy is defined here as the number of sites at which a taxon was recorded. The 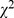 test of independence was used to test for a relationship between depth (shelf vs. slope) or phylum and the relative abundance of rare taxa. Relative abundance was defined as the average contribution of a taxon to the total number of individuals in each sample where the taxa were present. The Wilcoxon rank sum test was used to assess the significance of shelf–slope differences in total abundance, taxa richness (number of taxa) and taxonomic distinctness [46]. A measure of evenness was not included in our analysis as evenness was constrained as a result of low counts and taxa richness at several stations (see [47]). To differentiate between possible drivers of variation in abundance and taxa richness across depth and sampling years a two-way analysis of variance was used. Longitude and latitude were not included in this analysis as they were correlated with depth and year. Individual-based rarefaction curves were used to investigate the degree to which sample size and mesh size differences affected the abundance and taxa richness patterns across sample years.

Multivariate cluster and ordination techniques were utilized to explore the macro- and megafaunal assemblage patterns. A fourth-root transformation was applied to the matrices to reduce the influence of highly abundant taxa [48]. The Bray-Curtis (BC) dissimilarity measure was computed to obtain an ecologically meaningful distance measure based on the relative abundance and composition of taxa between stations. 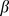 diversity was computed using BC similarity (BC dissimilarity 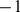). Ward's method of hierarchical clustering was used to define compact clusters of stations. The number of clusters was determined by selecting the maximum average silhouette width (ASW), a measure of average dissimilarity of stations within versus between clusters [49], for all combinations of cluster sizes. Station dissimilarities were also visualized through non-metric multidimensional scaling (nMDS) ordination. Average relative abundances of taxa, the contribution of each taxa to total abundance, by cluster were used to define dominant taxa representative of clusters.

## Results and Discussion

### Distribution of occurrence, abundance and rarity

Two hundred and forty-seven taxa were collected at the 48 stations sampled. A total of 4,752 individuals sampled from a 6 m^2^ area were distributed among 80 macrofauna taxa and a total of 452,115 individuals sampled from a 21,600 m^2^ area (approx.) were distributed among 167 megafauna taxa. Piepenburg et al. [30] estimated the Beaufort Shelf holds around 1,100 species of major macro- and megafaunal taxa (annelids, arthropods, echinoderms and molluscs), which suggests we have only captured roughly one quarter of the taxa present. Most of the abundance was concentrated in polychaeta (66%), malacostraca (15%) and bivalvia (10%) classes in the macrofauna and ophiuroidea (28%), malacostraca (23%) and asteroidea (13%) classes in the megafauna. Many taxa had very low frequencies of occurrence. The macrofauna had 24 uniques (30%) (taxa present at only one station) and the megafauna had 32 uniques (19%), slightly lower compared to other continental shelves (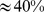) [26], [50] but similar to other Arctic regions (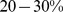) [25], [51]. The true percent of uniques may be higher, considering that the number of uniques was likely deflated by the grouping of many taxa to the family taxonomic rank.

**Figure 2:**
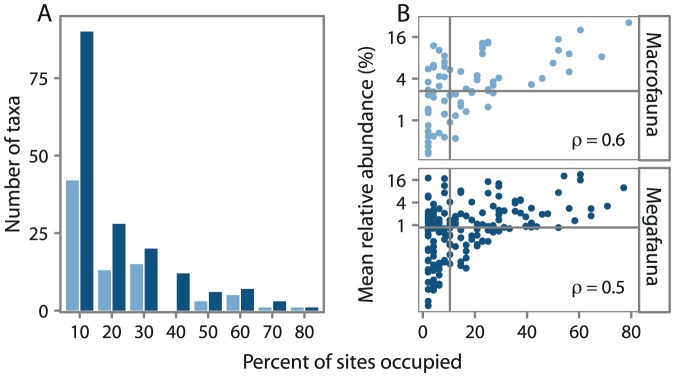
Distribution of occurrence. (A) Distribution of occurrence as percent of sites occupied (binned intervals starting with 1-10%) and (B) mean relative abundance (%) by percent of sites occupied. Relative abundance, a measure of local abundance, was averaged only across sites where taxa were present. Vertical grey line represents rarity cut-off at 10% and horizontal grey line denotes the median average relative abundance. Spearman's rank correlation coefficient denoted by 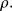

Beaufort macro- and megafauna displayed the typical right-skewed distribution of occurrence [52], where most taxa are rare and few are widespread (Figure 2A). Rare taxa are defined here as taxa restricted in occurrence (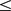 10% of stations), not necessarily in abundance. A property of this distribution is that rare taxa comprise a larger portion of total taxa richness at the regional scale (all samples) with a ratio of rare to common taxa of 1.3∶1 for macrofauna and 1.2∶1 for megafauna while at the sample scale common taxa comprise the largest portion of total taxa richness with a ratio of common to rare taxa of 6∶1 for macrofauna and 8∶1 for megafauna. At all scales, rare taxa comprise a greater proportion of total macrofaunal taxa. However, this may simply be an artifact of differences in sample area. The larger sample area of trawls makes them more likely to collect patchy and sparsely distributed taxa. Previous studies have demonstrated a positive correlation between the presence of rare taxa and depth [51]; however, we found no such relationship when taking into account proportion (data not shown). Rather, we found the number of rare taxa was a function of the total taxa richness (macrofauna: Spearman's 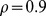, 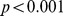; megafauna: Spearman's 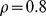, 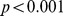), similar to the findings of Etter and Mullineaux [53]. Additionally, though rarity can be dependent of phyla in terrestrial systems [54], [55], we were not able to reject the null hypothesis that rare taxa were distributed with equal proportion among phyla (data not shown).

**Figure 3:**
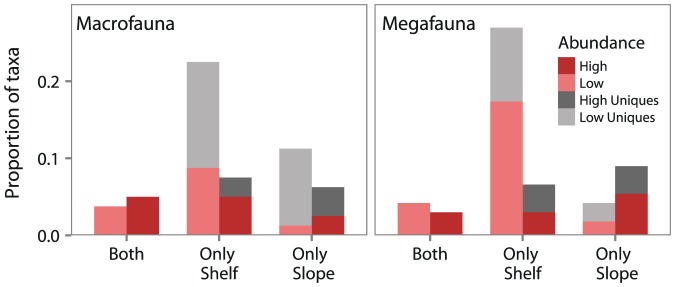
Proportion of rare taxa unique to or shared between shelf and slope. The proportion of low or highly abundant rare taxa sampled only on the shelf, slope or both localities. Rare taxa defined as taxa occurring at 10% of sites or less. Uniques (taxa which were sampled only at one site) are distinguished from other rare taxa. High and low relative abundance defined as greater or lower than the median.

A positive relationship exists between occupancy and average relative abundance (Figure 2B) for both faunal groups (macrofauna: Spearman's 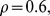 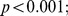 megafauna: Spearman's 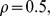 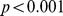). As expected, common taxa tended to be higher in local relative abundance than rare taxa which on average contributed less to total abundance per station [56]. However, some rare taxa were relatively abundant at the few stations they were present (ex. macrofauna: *Pseudosphyrapus serratus*, Thyasiridae, *Terebellides*; megafauna: *Apomatus similis*, Pectinidae, *Siphonodentalium*, *Ophiura*). These rare taxa, high in relative abundance, may be habitat specialists dominant in their niche but unable to persist in other habitats [26], [57]. Or, their abundance may be the result of a localized disturbance or recruitment event. Alternatively, these taxa may be pseudo-rare: taxa that appear rare because they are sampled on the fringe of their optimal depth range [58], [59] and thus were only present in larger numbers in samples from favourable depths (ex. deep-sea taxa such as *Pseudosphyrapus serratus* and *Siphonodentalium*). Pseudo-rarity is the only testable hypothesis with the available data. To determine the likelihood that pseudo-rare taxa were present we examined whether highly abundant rare taxa (greater than the median) were more likely to be restricted to the shelf, slope or present on both than rare taxa that were low in abundance. Uniques were not considered in this analysis. We found a greater proportion of low in abundance rare taxa were restricted to the shelf and a greater proportion of highly abundant rare taxa were restricted to the slope (Figure 3), however the difference was only statistically significant for megafaunal taxa (macrofauna: 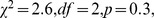 megafauna: 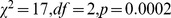). This suggests that rare slope taxa are more likely to be pseudo-rare while rare shelf taxa may be restricted in occurrence due to sparsity (low population size).

### Patterns in abundance and taxa richness

**Figure 4:**
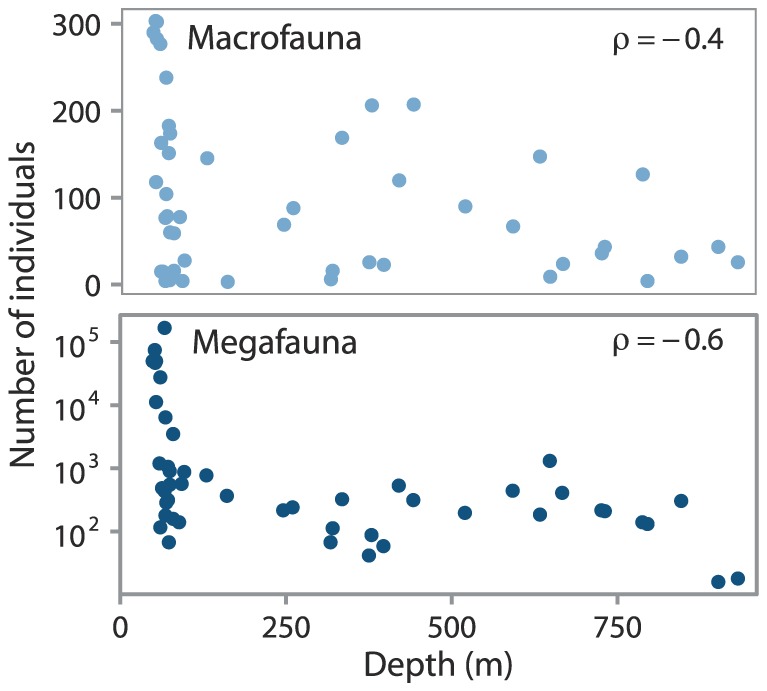
Relationships of total macro- and megafaunal abundance with depth. Abundance in number of individuals per sample. Sample area of macrofauna: 0.125 m^2^ and megafauna: 450 m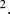 Spearman's rank correlation coefficient denoted by 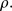

**Figure 5:**
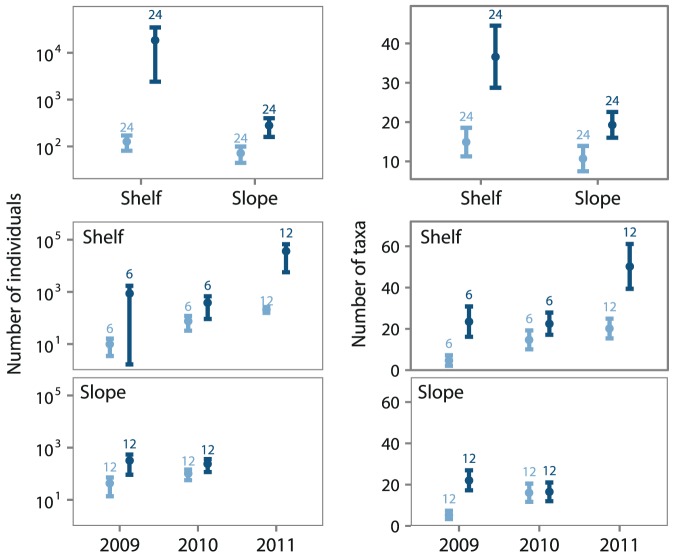
Comparison of macrofaunal (light blue) and megafaunal (dark blue) abundance and taxa richness between shelf and slope stations and sampling years. Mean total abundance (left panel) and mean taxa richness (right panel). Stations grouped by shelf and slope (top panel) and stations grouped by year on shelf or slope (bottom panels). Bars represent 95% confidence intervals. Sample size (N) is denoted by number on bar. Sample area of macrofauna: 0.125 m^2^ and megafauna: 450 m
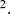

Total abundance on the Beaufort shelf decreased with depth (Figure 4), confirming Conlan et al.'s [23] result. Megafauna showed a stronger negative correlation between abundance and depth (megafauna: Spearman's 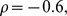 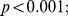 macrofauna: Spearman's 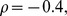 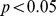) and had a larger range of abundance values on the shelf than slope (F-test: 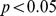). Yet, no decrease in abundance with depth was observed when the depth range was restricted to the slope between 100 to 1,000 m (macrofauna: Spearman's 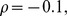 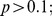 megafauna: Spearman's 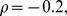 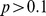). Differences between macro- and megafauna in abundance and taxa richness (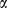 diversity) across the depth gradient are illustrated by grouping shelf and slope stations (Figure 5). Macrofaunal shelf stations showed slightly greater mean abundance and mean richness compared to slope stations, but this difference was not statistically significant (Wilcoxon test: 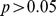). Megafauna were on average significantly more abundant and taxa rich (Wilcoxon test: 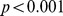) at shelf stations than at slope stations. Renaud et al. [14] showed similar declines in larger fauna with depth on the Beaufort Shelf. Declines in megafaunal abundance while macrofauna remain relatively constant across the depth range (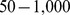 m) supports the notion of increased prevalence of smaller body sizes with depth [16], [60]–[63]. The most parsimonious explanation for the observed shift from larger to smaller size classes with depth is the diminishing supply of organic material [64] as larger fauna require more energy to survive and reproduce [15], [16]. No difference in taxonomic distinctness was found between shelf and slope for macro- or megafauna (Wilcoxon test: 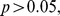 data not shown).

Shelf–slope differences are partially confounded by temporal and spatial variability introduced through the multiple year sampling scheme, 2009 to 2011. Over this time period, the spatial extent of sea-ice decreased, sampling was carried out further into the ice-free season and farther to the east (Figure 1). To illustrate the potential effects of this temporally associated variability in macro- and megafaunal abundance and taxa richness, shelf and slope stations were grouped by sampling year (Figure 5). Variation in macrofaunal abundance and richness was explained by both depth and year with year explaining more of the total variance (Table 1). Macrofaunal mean abundance and richness increased each year regardless of position on shelf or slope. Variation in megafaunal abundance and richness was also explained by both depth and year, however, depth explained more of the total variance (Table 1). We found a significant interaction between depth and year which indicates the affect of year on megafaunal abundance and richness was not consistent across shelf and slope stations. This interaction may be an artifact of inconsistencies in trawl sample areas (discussion below) in combination with the lack of slope stations in 2011.

**Table 1:** Analysis of variance of macro- and megafaunal abundance and taxa richness with year and depth.

	Response	Source	MS	F	*p*
**Macrofauna**	Abundance	Depth	2.7	3.6	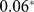
		Year	48	64	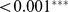
		Depth x Year	0.3	0.4	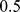
	Richness	Depth	210	6.6	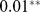
		Year	1600	50	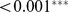
		Depth x Year	45	1.4	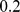
**Megafauna**	Abundance	Depth	65	27	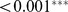
		Year	35	15	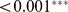
		Depth x Year	17	7.2	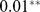
	Richness	Depth	3600	28	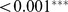
		Year	1900	15	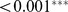
		Depth x Year	1800	14	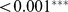

Categorical variables: depth  =  shelf/slope and year  =  2009/2010/2011. Abundance was log transformed to normalize residuals. Significance codes: 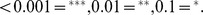

Spatial location is likely to influence the distribution of abundance and taxa richness as a high degree of benthic spatial heterogeneity exists on Arctic shelves [24], [65]. In addition, seasonal and temporal variability of Arctic benthos across multi-year sampling programs have been found to be insignificant relative to spatial variability (V. Roy and P. Archambault, unpublished data). Therefore, the variance explained by sampling year in our analysis (Table 1) is more likely a result of location. As sampling occurred farther to the east with each subsequent year, greater macrofaunal abundance and richness may be a consequence of the proximity to nutrient rich upwelled water from Cape Bathurst [42].

**Figure 6:**
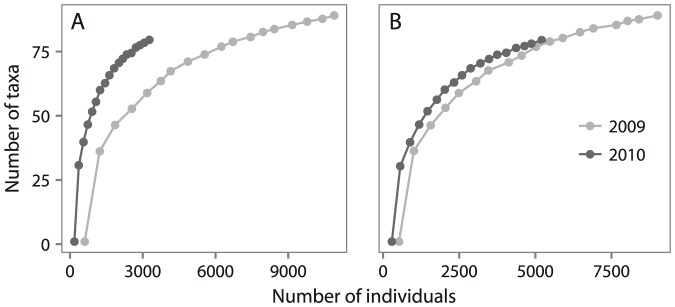
Individual-based rarefaction curves for 2009 and 2010 megafaunal datasets. (A) non-normalized counts and (B) counts normalized to the average trawl area. Curves represent the average of 900 resampling permutations.

If the location of sampling was indeed affecting abundance and richness patterns, why were megafaunal abundance and richness similar in 2009 and 2010 (Figure 5)? Abundance and richness values from 2009 trawls may be inflated as a consequence of the larger sample size and smaller mesh sieve utilized that year (described in Methods). A larger sample size collects more individuals and a sieve with a smaller mesh retains more juveniles and small bodied species, thereby inflating the total number of individuals and taxa present in the sample [66]. Taxa richness can be further affected by sample size differences because it cannot be normalized to a standard sample size as richness does not vary linearly with sample area [66]. Normalizing total abundance by a standard sample size (utilized in this study) controls for the increase in individuals from the larger sample but not for the smaller mesh. In addition, normalizing counts to a standard sample size can be problematic when comparing taxa richness between samples. Normalization reduces the taxa-per-individual ratio for smaller sample sizes while increasing the ratio for larger sample sizes, in this case artificially bringing the 2009 and 2010 taxa-per-individual ratios and thus rarefaction curves closer together (Figure 6B). However, non-normalized individual-based rarefaction curves demonstrate that taxa richness in 2010 was actually higher than in 2009 when measured at comparable abundances (Figure 6), which agrees with our observation that richness increased with each sampling year as stations moved eastward along the shelf and slope.

### Patterns in 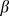 diversity

**Figure 7:**
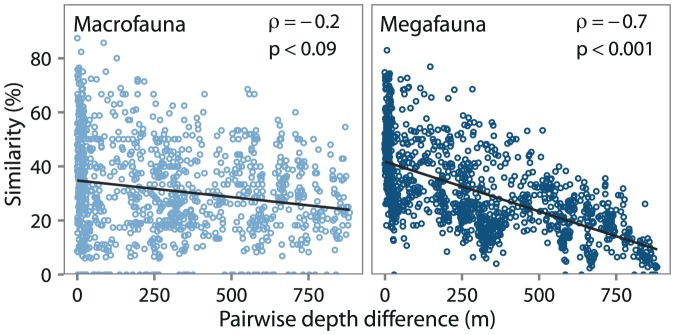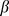 diversity across the depth gradient. 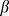
 diversity as a comparison of Bray-Curtis similarity between each pairwise depth difference. Spearman's rank correlation coefficient and significance (p-value) denoted by 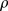 and p, respectively.

Macro- and megafauna differed in 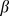 diversity patterns. Sixty-five percent of macrofaunal and only 46% of megafaunal taxa occurred on both the shelf and slope, not including uniques. Macrofauna shelf and slope taxa were similar in overlap to that previously observed at the pan-Arctic level (61%) [22], [30]. A significant negative correlation between community similarity and depth was only detected in the megafauna (macrofauna: Spearman's 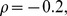 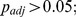 megafauna: Spearman's 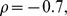 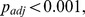 Figure 7), corroborating previous work that established megafauna had a faster rate of species replacement than macrofauna [32]. However, both species replacement and nestedness can drive 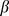 diversity patterns [67]. Nestedness, contrary to species replacement, is caused by species loss without a gain of new species along a gradient [68]. As described in the previous section, total abundance and taxa richness of the megafauna decreased more rapidly with depth. Considering that 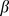 diversity is not independent of 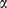 diversity [31], megafaunal 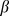 diversity could be purely driven by decreased 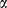 diversity with depth. Additionally, decreasing richness with depth could indicate that megafaunal 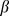 diversity is more likely driven by faunal loss (nestedness) than faunal replacement.

**Figure 8:**
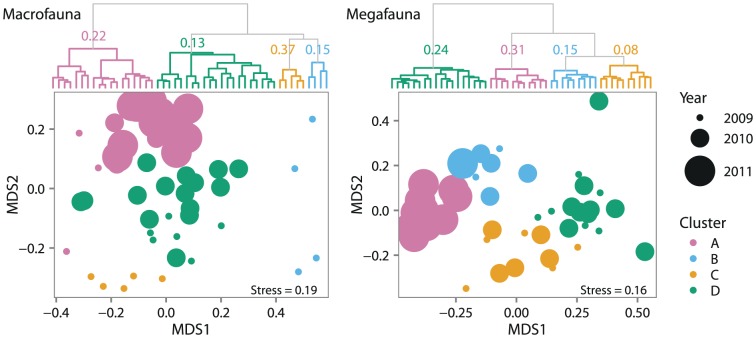
Dendrogram and nMDS ordination of station similarities. Hierarchical, Ward's method cluster dendrogram (top) and nMDS ordination highlighting clusters and sampling year (bottom) both derived from Bray-Curtis dissimilarity of macrofaunal and megafaunal abundance matrices. Average silhouette widths (scale 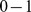) noted atop each cluster. Coloured circles in ordination represent macro- and megafaunal clusters defined in dendrograms. Circle sizes correspond to sample years indicated on right.

**Figure 9:**
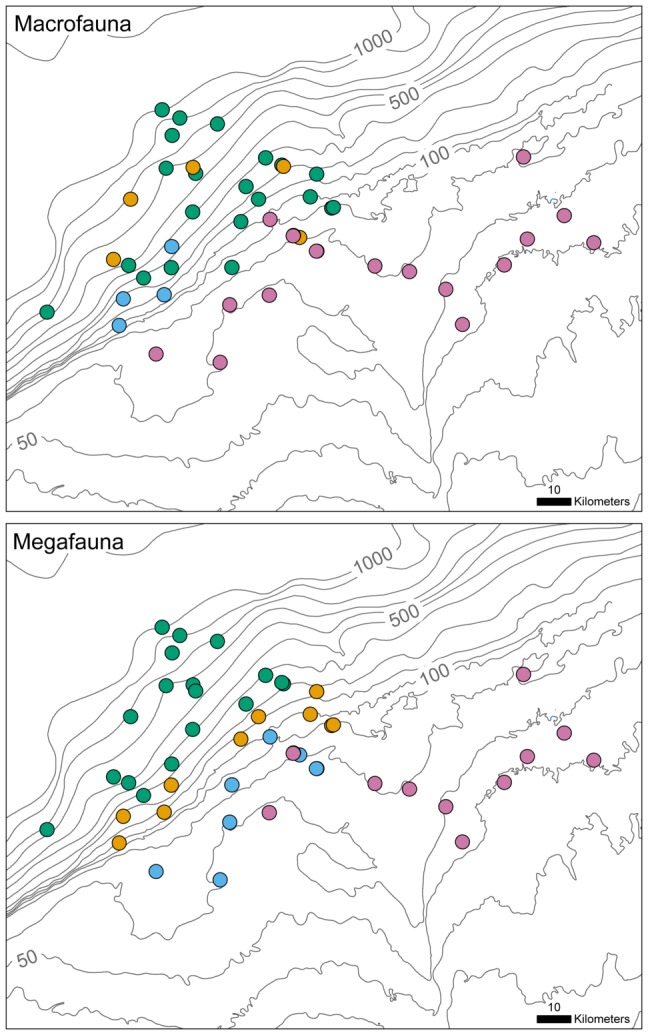
Map of Beaufort sampling region with georeferenced clusters. Colours represent macro- and megafaunal clusters defined in dendrograms (Figure 8).

To distinguish between replacement and nestedness drivers of 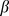 diversity, stations were clustered based on similarity in composition and relative abundance of taxa (Figure 8). Mean average silhouette widths (ASW), a measure of between versus within cluster variability at a scale from 0 to 1, were low for macrofauna (0.19) and megafauna (0.20) clusters. Low ASW is an indication that clusters represent loose groupings rather than distinct, structured assemblages [69], which fits the established view that faunal change is continuous across the depth gradient lacking distinct zones [15], [70]. The spatial distribution of clusters (Figure 9) depicts the bathymetric gradient as the major structuring factor in station clustering. In agreement with the 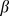 diversity results, megafaunal clusters were more clearly distributed according to depth. Megafaunal groupings on Arctic shelves have previously been noted to follow depth gradients [71], [72], likely shaped by food availability [70], [73], [74]. Sediment properties were not likely a major cause of the faunal clustering as sediment grain size on the Beaufort Shelf does not vary largely with depth [14], but more so along the east-west axis [75]. Different water masses found on the shelf and slope are also unlikely to be shaping the bathymetric trends. Shelf water of mainly Pacific origin and slope water of mainly of Atlantic origin (>200 m) have relatively little variation in salinity (32 to 34‰), temperature (−1.5 to 0.5°C) [37], [38] and dissolved oxygen (6 to 7 ml L^−1^) [23], [76].

The superposition of clusters and sampling year on macro- and megafaunal stations in ordination space illustrates the potential contribution of sampling year and location (as stations were farther to the east each year of sampling) to station similarities (Figure 8). Qualitatively, macrofaunal clusters were more likely shaped by sampling year and/or location than megafaunal clusters as stations that clustered together were more likely to be from the same year in the macrofauna than the megafauna. That observation is supported by our finding that macrofauna have a stronger negative correlation between community similarity and year (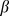 diversity across sampling years) than megafauna (macrofauna: Spearman's 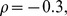 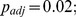 megafauna: Spearman's 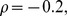 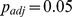).

**Figure 10:**
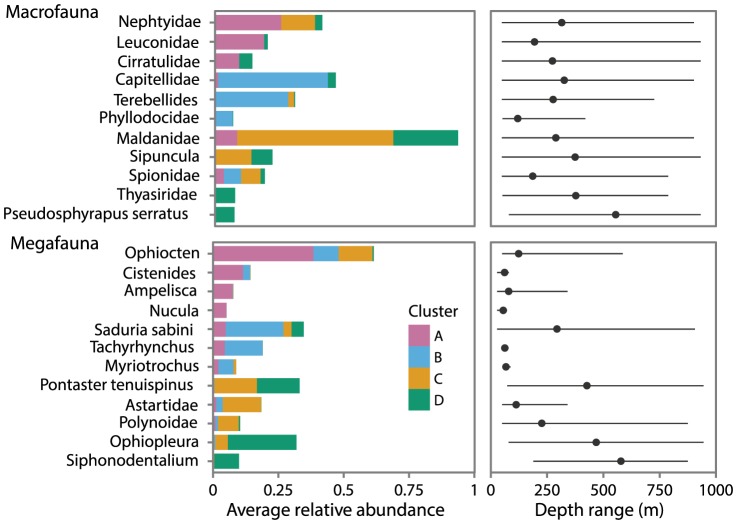
Relative abundance and depth ranges of dominant taxa. Average relative abundance of dominant taxa by cluster (left). Dominant taxa ordered by their contribution to average relative abundance in clusters A, B, C and D, are highlighted by cluster to illustrate the relative contribution of taxa to each cluster. Colours represent faunal clusters defined in dendrograms (Figure 8). Depth range of corresponding taxa (right); grey circles denote mean depth of samples where taxa were present.

Changes in the dominant macro- and megafaunal taxa between clusters reveal compositional differences (Figure 10A) that provide evidence of faunal replacement as a main driver of 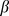 diversity. Dominant taxa were defined as the top four taxa in terms of average relative abundance for each cluster. The dominant taxa typically represented over 50% of the cumulative average relative abundance per cluster (macrofauna: A = 62%, B = 83%, C = 94%, D = 48%; megafauna: A = 63%, B = 52%, C = 52%, D = 57%). Across all clusters, the macrofauna were principally comprised of polychaetes. Macrofaunal cluster A (shelf cluster) was characterized by cirratulid and nephtyid polychaetes and leuconid cumaceans. Macrofaunal cluster D (slope cluster) was characterized by maldanid polychaetes, Sipuncula, thyasirid bivalves and tanaid *Pseudosphyrapus serratus*. Clusters B and C, also found on the slope but compositionally distinguishable from D cluster macrofauna, may be influenced by smaller scale processes and forces than depth gradients. Cluster B was distinct in having higher relative abundances of polychaetes Capitellidae, *Terebellides* sp. and Phyllodocidae compared to other slope clusters. Cluster C was distinguished by its extremely high relative abundance of maldanid polychaetes (60% of total abundance on average).

Across all clusters, the megafauna were principally comprised of echinoderms, typical of Arctic shelves [10]. Megafauna shelf cluster A, containing only 2011 stations, was characterized by the ophiuroid *Ophiocten* sp. as well as polychaete *Cistenides* sp., amphipod *Ampelisca* sp. and bivalve *Nucula* sp.; shelf cluster B, located west of cluster A and Kugmallit Valley, was characterized by isopod *Saduria sabini*, gastropod *Tachyrhynchus* sp. and holothurian *Myriotrochus* sp. Megafauna shelf break cluster C was characterized by asteroid *Pontaster tenuispinus*, astartid bivalves, ophiuroid *Ophiocten* sp. and polynoid polychaetes. Megafauna slope cluster D was characterized by ophiurid *Ophiopleura* sp., asteroid *Pontaster tenuispinus* and scaphopod *Siphonodentalium* sp. Some megafauna taxa dominant in shelf clusters were not present on the slope and vise versa (Figure 10B) providing evidence that slope taxa are not just a nested subset of shelf taxa.

## Conclusions

The Canadian Beaufort shelf and slope host a diverse assemblage of macro- and megafauna, 247 taxa−many (30%) of which were classed at the family taxonomic rank or higher, indicating species richness may be much greater. At a regional scale, rare taxa constituted the majority of taxa represented while faunal numbers in individual samples were dominated by taxa common throughout the region. We found that the proportion of rare taxa was independent of depth and phylum, instead rarity was simply a function of the total taxa richness––the more taxa present in a sample the greater the number of rare taxa. We hypothesize that rare taxa are driven by different processes on the shelf (sparsity) and slope (pseudo-rarity), since rare taxa on the slope tended to contribute more to local faunal numbers compared to those on the shelf.

Our results indicate that macro- and megafauna have divergent patterns of abundance and 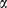 and 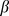 diversity on the Beaufort shelf and slope. Macrofauna showed greater change in 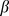 diversity with year and/or location compared to the megafauna, although this relationship was weak for both faunal groups. Megafauna show greater change in abundance, taxa richness and 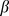 diversity with depth compared to the macrofauna, conceivably owing to a greater cost of the declining food supply for larger bodied organisms. We infer that megafaunal 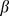 diversity is not merely driven by 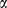 diversity, since shifts in dominant taxa are evident between shelf and slope clusters. Furthermore, we deduce that faunal replacement is a greater driver of megafaunal 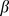 diversity than nestedness as some dominant megafauna in shelf clusters are not present on the slope and vice versa, suggesting that slope taxa are indeed differentiated from shelf taxa and not solely a nested subset of shelf taxa.

The effect of temporal variability on the benthos is less clear. Sampling year explained a portion of both macro- and megafaunal variability in abundance and richness, however, year effects were highly confounded by location on the shelf and the timing of sampling with respect to sea-ice conditions (with sampling occurring later into the summer season with each year). We speculate that the major source of this temporally associated variability was spatial heterogeneity; however, it may be a combination of several factors such as a longer growing season, temporally variable (spring vs. summer) recruitment events or faster growth with increased primary productivity input to the benthos under reduced ice conditions. The latter would support the notion that the benthic standing stock will increase as sea-ice retreats in the Arctic. Under the alternative scenario, the loss of sinking ice-algae and a shift toward open-water primary productivity leads to a decreased food supply for shelf benthos. If this scenario holds true on the Beaufort shelf, our observations suggest that shelf fauna could become more similar to the present day slope fauna. As slope megafauna are differentiated from shelf fauna and smaller body size organisms (macrofauna) contribute more to total abundance on the slope, this would likely have cascading affects on higher trophic levels [77], [78].

As development encroaches and the Beaufort shelf and slope are exposed to pressures from industry and climate warming, further benthic monitoring will be essential to the management of biodiversity and ecosystem services. The establishment of effective long-term monitoring in the region will require a better understanding of larger scale spatial differences along the shelf. Several questions remain for future research: Are bathymetric trends in macro- and megafauna diversity and community structure consistent from east to west along the shelf? How might scale affect the observed spatial patterns? How does the magnitude of spatial variability in faunal abundance compare to temporal and depth related variability? Answering these questions will be crucial to distinguishing long-term changes in the benthos from spatial and interannual variability.
